# Supplementary material for: Evidence supported by Mendelian randomization: impact on inflammatory factors in knee osteoarthritis
Source: Front Med (Lausanne). 2024 May 28;11:1382836. doi: 10.3389/fmed.2024.1382836 (PMC11165061; doi:10.3389/fmed.2024.1382836)
Supplement: Supplementary file 1 [file Data_Sheet_1.ZIP › data/GCST90274800_5e6/800.forest.pdf]

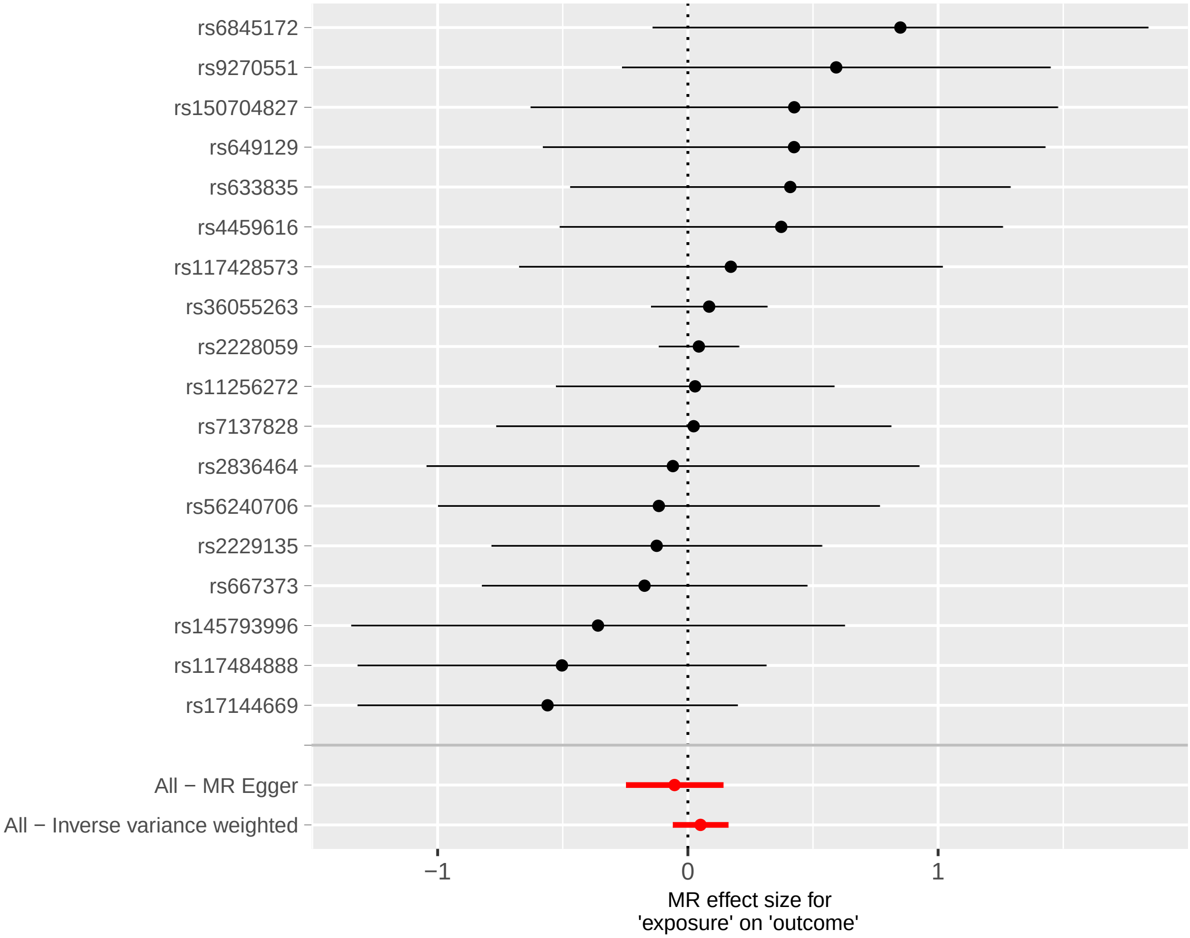
rs6845172
rs9270551
rs150704827
rs649129
rs633835
rs4459616
rs117428573
rs36055263
rs2228059
rs11256272
rs7137828
rs2836464
rs56240706
rs2229135
rs667373
rs145793996
rs117484888
rs17144669
All − MR Egger
All − Inverse variance weighted
−1
0
1
MR effect size for
'exposure' on 'outcome'
